# Increasing the Strength and Production of Artemisinin and Its Derivatives

**DOI:** 10.3390/molecules23010100

**Published:** 2018-01-03

**Authors:** Syed Lal Badshah, Asad Ullah, Nasir Ahmad, Zainab M. Almarhoon, Yahia Mabkhot

**Affiliations:** 1 Department of Chemistry, Islamia College University Peshawar, Peshawar 25120, Pakistan; asadullah@icp.edu.pk (A.U.); nasirshah121@yahoo.com (N.A.); 2 Department of Chemistry, College of Sciences, King Saud University, Riyadh 11451, Saudi Arabia; zalmarhoon@ksu.edu.sa

**Keywords:** artemisinin, novel analogues, malaria, Plasmodium, mass scale production

## Abstract

Artemisinin is a natural sesquiterpene lactone obtained from the *Artemisia annua* herb. It is widely used for the treatment of malaria. In this article, we have reviewed the role of artemisinin in controlling malaria, spread of resistance to artemisinin and the different methods used for its large scale production. The highest amount of artemisinin gene expression in tobacco leaf chloroplast leads to the production of 0.8 mg/g of the dry weight of the plant. This will revolutionize the treatment and control of malaria in third world countries. Furthermore, the generations of novel derivatives of artemisinin- and trioxane ring structure-inspired compounds are important for the treatment of malaria caused by resistant plasmodial species. Synthetic endoperoxide-like artefenomel and its derivatives are crucial for the control of malaria and such synthetic compounds should be further explored.

## 1. Introduction and Background

Malaria is one of the devastating diseases affecting millions of people across Asia and Africa each year. It is caused by the protozoan Plasmodium and the vector is the *Anopheles* mosquito. The mortality rate from malaria was reduced and a great success was achieved, however due to the lack of funds, malaria is on the rise again as reported by the World Health Organization [1]. There were approximately five million more cases of malaria in 2016 than the year before and the 40% reduction in malaria target set for the year 2020 is difficult to be achieved [1]. If this lack of funding for malaria control continues in the coming years then the sustainable development goals (SDGs) set for 2030 will be just a dream [2]. The world health organization reported that approximately 445,000 deaths occurred due to malaria in 2016, like the previous year [1,3]. The main controlling agents for malaria are, sleeping inside nets, spraying insecticides on the house walls and the use of artemisinin-based combination therapies (ACTs) as antimalarial agents [1]. Furthermore, in Southeast Asia a new species of Plasmodium called *Plasmodium knowlesi* has emerged that is mostly misdiagnosed and its malarial death toll is increasing day by day [4]. The death toll is about one million every year from malaria in children under the age of five in African countries [5]. On a global scale, it is a big challenge that malaria can be eliminated completely and in a majority of countries implementation of strategies for elimination of malaria has been started [6], while it is also a fact that the present available drugs are not sufficient to eliminate malaria completely [7]. There is a need for safe single dose therapies that are also suitable for mass drug administration to asymptomatic carriers and capable of blocking malaria transmission through the *Anopheles* mosquito vector. In addition, chemoprophylaxis prevention requires drugs that are able to eliminate the liver stage forms of the parasite (especially for *Plasmodium vivax*). The emerging threat of resistance to artemisinin drugs forced philanthropic organizations like Welcome Trust of England, Bill and Melinda Gates foundation, as well as the establishment of public private partnerships to take concrete steps for discovery and development of novel antimalarial drugs [8,9,10,11]. Biomedical research work in different laboratories is underway to discover and develop such drugs which are more efficacious against malaria. These drugs will target multiple stages of the life cycle of the plasmodium, help in prevention, provide immediate cure and thus block its transmission [12]. The four species of protozoan parasites of plasmodium mainly causing malaria include *Plasmodium falciparum*, *P. vivax*, *P. malariae*, and *P. ovale*, while the transmission is carried out by over 70 species of *Anopheles* mosquitoes [13,14]. The *P. falciparum* and *P. vivax* are the most prominent species among all that cause malaria. About 90% of deaths due to malaria that are reported from Africa and Asia are due to *P. vivax*. Similarly, *P. vivax* is also responsible for malaria in the Middle East, western Pacific and in central and south America, however *P. vivax* is less lethal than the *P. falciparum* [15]. Majority of the malarial cases and deaths are associated with the *P. falciparum*. The growing resistance to chloroquine and limited use of artemisinin analogs are responsible for the dire need of discovery of novel antimalarial agents. All the processes of malaria, including life cycle, immunological defense mechanisms, and the clinical development of malaria are complex in nature. Periodic fever is related to clinical malaria and after the breakage of the infected erythrocytes that occur due to the induction of the cytokines interleukin-1 and tumor necrosis factor. Malaria caused by *P. falciparum* can have devastating effects, like anemia, cerebral complications (from coma to convulsions), hypoglycemia and glomerulo-nephritis, and it is more serious in non-immune people including children, tourists and pregnant women. For such a serious disease, there are few remedies in the form of herbs that contain important chemical compounds and since ancient times these medicinal plants like *Cinchona succiruba* and others are used for the treatment of malaria [16,17,18,19,20]. The current available effective therapies for malaria include the combination of artemisinin with other drugs. Artemisinin is the sesquiterpene alkaloid present in the aerial parts of the Asian plant of *Artemisia annua* L [21]. *Artemisia annua* herb commonly called “sweet wormwood” belongs to the Asteraceae family [22]. It was initially isolated by the Chinese scientist Tu Youyou in 1972 from Artemisia herb and used as an antimalarial drug against *Plasmodium* [22]. For this discovery, Tu Youyou got the 2015 Nobel Prize in Physiology or Medicine [23,24]. Artemisinin can be used as an antimalarial, anticancer and anti-inflammatory agent [25,26,27,28]. The artemisinin and its derivatives also possesses anthelmintic, fungicidal and antiviral properties [29].

**Figure 1:**
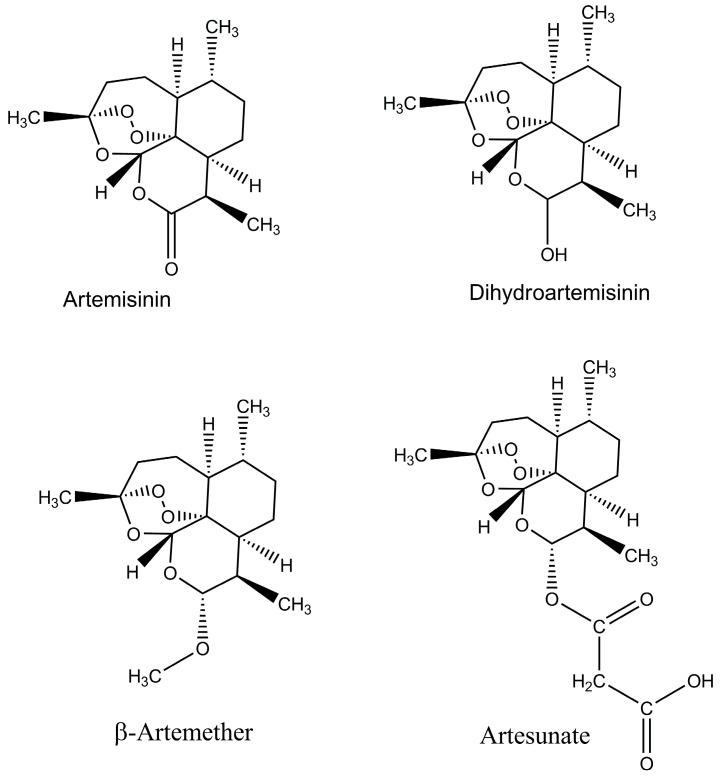
Chemical structure of artemisinin and its derivatives.

In the last ten years, the worldwide use of the Artemisinin-based combination therapies (ACTs) resulted in the reduction of global malaria morbidity and mortality. The WHO recommended the ACTs as a choice for the treatment of the severe and uncomplicated *P. falciparum* malaria in all areas where malaria is endemic [3,30], and they have been adopted as first-line therapy in many countries. Around 409 million ACT treatments were done in 2016 [1]. The following drugs are included in the artemisinin group: artesunate, artemether, and dihydroartemisinin are the most common, and these drugs have significant antimalarial activity and have the least side effects in patients (Figure 1). One issue is with the half-life of artemisinin; it has short half-life (<1 h) and about 7–10 days are required to achieve the high rate cure when it is used in its own way and has impact on adherence. It is the main reason to use the artemisinin in combination with long-acting partner drugs like lumefantrine, amodiaquine and piperaquine. As result of this combination, a cure can be achieved within three days of treatment, while the combination of artemisinin and lumefantrine is used throughout the world and is very effective [31]. For the treatment of malaria, especially among children, it is used in the combination form of Artesunate–amodiaquine (AS–AQ), but unfortunately, the early signs of resistance were observed in South East Asia which is alarming for the achievements gained in the control of malaria [32,33]. It has been observed that artemisinin resistance is mainly related with mutations in the *kelch13* gene [32,34,35], and the detection of this genetic marker will greatly enhance possible resistance surveillance [36,37]. Emerging resistance was initially identified as delayed parasite clearance rates following treatment with artemisinin-based therapies. Confirmed partial artemisinin resistance is now defined by the WHO as ≥5% of patients carrying K13 resistance-associated mutations, all of whom have been found, after treatment with ACT or artesunate monotherapy, to have either persistent parasitemia by microscopy on day three, or a parasite clearance half-life of ≥5 h [38,39]. Reflecting the importance of this issue, the WHO launched its Global Plan on Artemisinin Resistance Containment in 2011 with a specific emergency response to artemisinin resistance in the Greater Mekong sub-region in 2013 [40,41,42,43]. In addition, there are reports of reduced clinical efficacy of ACT therapy after 28 days of follow-up in some settings [38,39]. It is important to determine the extent to which this reduced efficacy reflects true resistance versus sub-optimal dosing, or other factors. The development of widespread resistance has limited the utility of numerous other antimalarials that were previously widely used, such as chloroquine and sulfadoxine–pyrimethamine, providing a sobering reminder of the potential impact of evolving resistance to drugs in current use [40]. With no new drugs immediately available to replace artemisinins, it is essential to optimize and define dosing strategies to ensure maximum therapeutic efficacy of ACTs, limit the spread of resistance and extend the clinical life of ACTs [44,45,46,47].

## 2. Increased Production of Artemisinin and Its Analogues

Cost and poverty burden is a big hurdle in controlling malaria, as artemisinin-based combination therapies (ACTs) are expensive. The content of artemisinin in its aerial parts is 0.01–1.2% while the annual world demand is around 119 metric tons (MT) [48] and it is not sufficient to fulfill the demand while total synthesis of artemisinin is too costly. Different approaches are used to get high content of artemisinin. The content of artemisinin in the *A. annua* plant was increased through metabolic engineering. In this regard, two different approaches are used; one is to overexpress the enzyme genes involved in the biosynthetic pathway of artemisinin, and the second is to block enzyme genes that are in competition with the artemisinin pathway from expression [49]. A number of studies have been done to elaborate the biosynthetic pathway of artemisinin and its expression in different laboratory subjects like *E. coli* and Baker’s yeast. In one strategy, yeast is exploited for the production of artimisinic acid by expression of the biosynthetic pathway of artemisinin [50,51,52]. This semisynthetic method is also expensive because of the cost of growth media and maintenance of yeast culture in bioreactors [52].

Paddon et al. developed a genetically modified strain of *Saccharomyces cerevisiae* by expressing the artemisinin pathway genes in this yeast [51]. The artemisinin production rate achieved was around 25 g/L of the fermentation media. However, there are a number of disadvantages; these include the costly media, isolation and purification of artemisinin from the fermenting media, etc. [51]. The efforts for increasing either artemisinin or its precursor production in yeast is still ongoing using different synthetic biological approaches [53]. In Brazil, where the production of sugar cane is high, it has been used for the production of semisynthetic artemisinin through yeast fermentation [54]. Initially, the fermentation of sugar cane juice is used for the production of β-farnesene, which is a sesquiterpene alkene [54]. The β-farnesene can then be chemically converted into artemisinin [54]. Thus, through this method of large scale production, the cost of artemisinin production can be lowered.

The use of transgenic tobacco plants for the production was initially tested by Zhang et al. who showed that the plant produces artemisinic alcohol, dihydroartemisinic alcohol and amorphadienes along with other related compounds. The transgenic tobacco plant produced the reduced form of the final product instead of the oxidized form [55]. These initial results showed that the tobacco plant can be modified to produce artemisinin. Farhi et al. took the same task and they expressed all the genes of the artemisinin and mevlonate pathway in tobacco plants using a single vector [56]. Although the production level of artemisinin was very low due to the production of side products, this paved the way for the use of tobacco for the production of this essential antimalarial drug [56].

**Figure 2:**
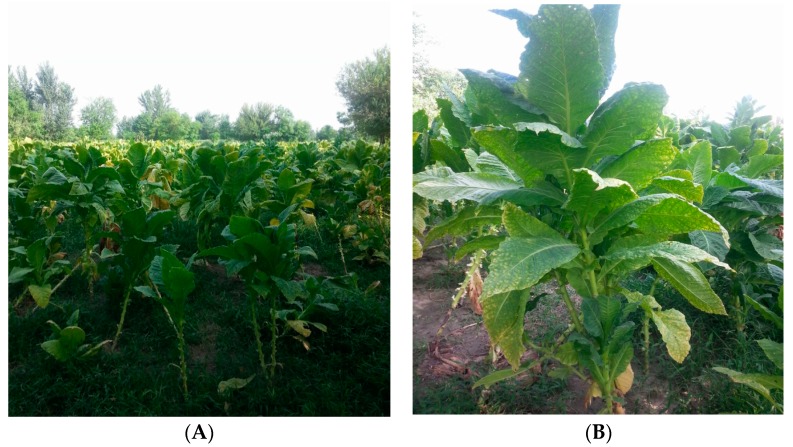
(**A**) Mature tobacco plants growing in the field of district Swabi, Pakistan; (**B**) The large size leaves of tobacco are suitable for the mass scale production of artemisinin.

A new synthetic biology approach was used by Bock and coworkers at the Max–Planck Institute, Germany, who were successful in expressing the whole enzymes of the artemisinin biosynthetic pathway inside the chloroplast of tobacco plants (Figure 2) [57]. They introduced the artemisinin full biosynthesis pathway genes into the chloroplast at once through their combinatorial supertransformation of transplastomic recipient lines (COSTREL) method [57]. The chloroplast is suitable for expression of foreign genes because the expression of genes is easy, foreign genes are easily accommodated due to the homologous recombination when foreign genes are transferred. They also introduced some accessory genes into the nucleus of the tobacco plant, however, their exact function is not known, but it is suggested that they are essential for the regulation of the artemisinin biosynthesis pathway. After transplastomy, the plant species were grown on spectinomycin so that they become homoplasmic (all plastid DNA copies contain the artemisinin biosynthesis genes). The plants developed the *Nt*-AO3-1 phenotype in which the artemisinic acid accumulates throughout the life span of the leaf. This phenotype was further improved to accumulate around 120.4 ± 42 mg per kg of fresh weight (FW) and was named *Nt*-AO3-CS180. The correlation studies of various transplastomic lines showed that the presence of the genes for the two enzymes, aldehyde dehydrogenase (*ALDH1*) and 1-deoxy-d-xylulose-5-phosphate reductoisomerase (*DXR*) are responsible for the high artemisinin content in phenotypes. The higher the expression of these two genes, the higher the content of artimisinic acid. There is no toxic effect on the chloroplast of leaves and only a 13% decrease in biomass was observed in the highest producing artemisinic acid phenotype plant lines as compared to wild type plant. They chose chloroplast instead of cytosol for the expression of artemisinin genes. The reason is that the chloroplast offers a better redox milieu for the quantitative conversion of artemisinic alcohol to artemisinic acid. Tobacco plant has large green photosynthetic leaves and thus the level of artemisinin produced will be in large amounts. The amount of artemisinin is high in photosynthetic parts (chloroplasts) in the plant. Beside a number of advantages in expression of artemisinin synthesis genes in tobacco, one disadvantage of tobacco is that of pest control and that it requires the use of costly pesticides. Therefore, these tobacco lines should be crossed with those tobacco plants that are resistant to pests, tall and produce many large leaves. The seeds of these tobacco plants should be shared with countries like Pakistan, Australia, and Zimbabwe etc. where tobacco is grown in large amounts and conditions are suitable for its growth. Pakistan’s north western regions like Swabi, Mardan and Charsada districts are particularly suitable for tobacco and their tobacco is exported to Europe and America. For the last few years, the growth of tobacco was dwindling due to lower purchase prices by multinational companies. Now, it is time again to expand the growth of tobacco. Thus, with low cost treatment of artemisinin, the world will be able to control malaria. Now the artemisinin resistant strains are also emerging rapidly [34,58] and therefore, a multidrug strategy should be used to control malaria. For this purpose, novel analogues of artemisinin should be designed that should be given to patients with other new drugs like ELQ-300 that can block the growth of the *Plasmodium* at different stages of its lifecycle inside the human body [59,60,61]. This COSTREL method should be advanced further in the future for development of other important medicinal alkaloids that are used for the treatment of cancer and viral diseases, so that the plight of these diseases can be controlled through the supply of cheap drugs for the poor population of the world.

Instead of just the chloroplast compartment of the transgenic tobacco plant, Malhotra et al. targeted three compartments that include chloroplast, nucleus and mitochondria for the expression of artemisinin pathway genes [62]. They developed a number of transgenic tobacco plants that produce artemisinin [62]. The most highly artemisinin-producing plant has a capacity of approximately 0.8 mg/g of the dry weight of tobacco leaf [62]. This is the highest amount of artemisinin production so far from transgenic plants. They also tested their tobacco plant leaves on mice infected with plasmodium and the recovery from malaria was fast [62]. Thus, they concluded that utilizing artemisinin in the encapsulated form inside the leaves will result in lowering the cost of its extraction and purification [62]. It was also proposed that expressing the artemisinin in lettuce leaves and its consumption in the raw form will further improve artemisinin production, its utilization and controlling malaria [62].

**Figure 3:**
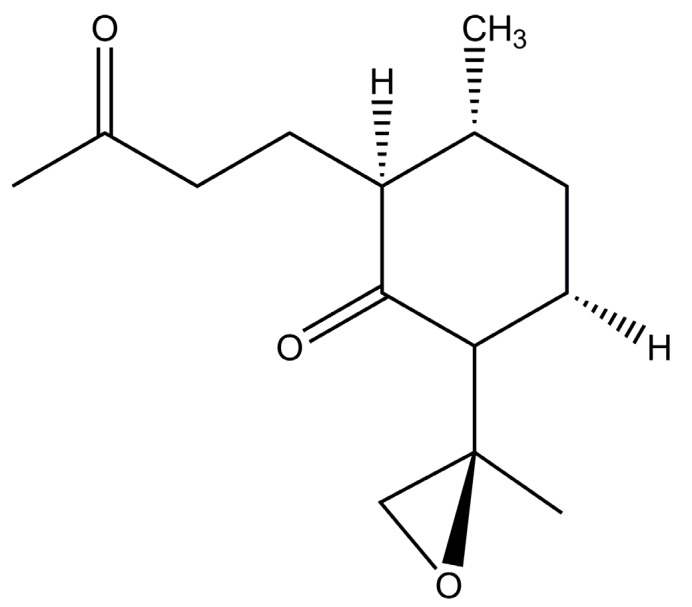
Chemical structure of arteannuin X [64].

Another approach is to increase the artemisinin content by increasing the trichome density content in the *A. annua* herb [63]. It has been observed that expressing the beta-glucosidase enzyme in *A. annua* herb results in a five-fold increase in artemisinin production due to increase in the trichomes density in leaves and flowers [63]. The metabolic pathway of artemisinin is quite interesting and it could be diverted for the production of related secondary metabolites. For example, Czechowski et al. found that introducing a mutation that inhibits the amorpha-4,11-diene C-12 oxidase (CYP71AV1) enzyme, which is required in several oxidation steps of artemisinin [64] resulted in the production of a sesquiterpene epoxide called arteannuin X and inhibition of artemisinin production (Figure 3) [64]. The complex trichomes of Artemisia plant can be explored for more potential sesquiterpenes through biochemical methods. It was also observed that when the *A. annua* is grown in vitro with beneficial bacteria like *Piriformospora indica* (Pi) and *Azotobacter chroococcum* (Az), the growth of the plant increases and the amount of artemisinin production doubles [65,66]. The boost in physiological and biochemical processes of *A. annua* through symbiosis is also a useful method for increasing the production of artemisinin.

**Figure 4:**
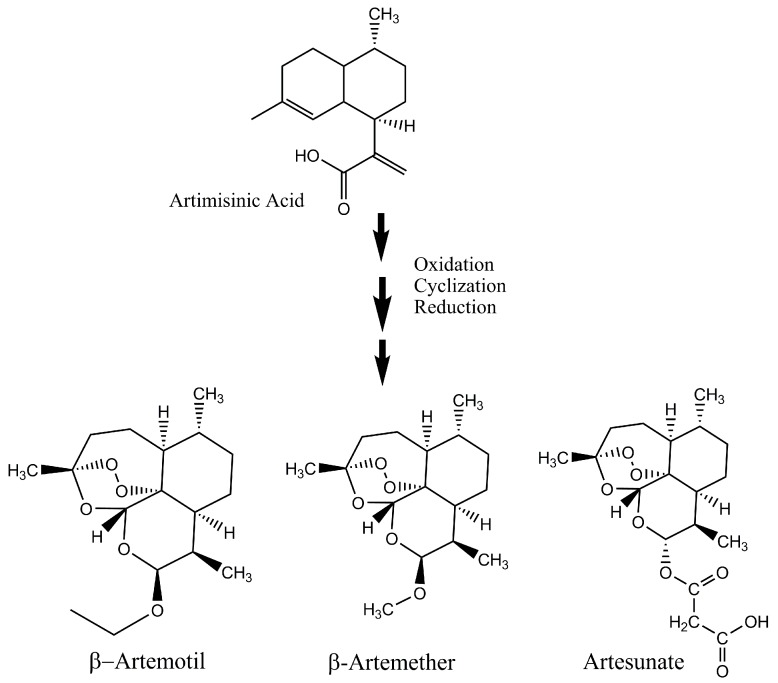
Production of active derivatives of artemisinin from artemisinic acid.

Singh et al. showed a novel method of artemisinin production from amorpha-4,11-diene that can easily be commercialized due to its favorable route of synthesis [67]. In this method, functionalization of the isopropenyl moiety of amorphadiene through *endo*-epoxyamorphadiene produced dihydroartemisinic acid [67]. This pure dihydroartemisinic acid is esterified, oxidized and its final cyclization resulted in artemisinin in high yields [67]. Some scientists have discovered short routes for the biosynthesis of artemisinin. The amorphadiene synthase, which is an important enzyme in the natural biosynthesis route of artemisinin [68], has the capability to transform oxygenated farnesyl diphosphate moiety straight away into dihydroartemisinic aldehyde. This aldehyde form of artemisinin can be converted into artemisinin in four simple steps into pure artemisinin, thus, making artemisinin from simple, easily accessible natural compounds into pure artemisinin through few simple steps is essential for the low-cost production of this important antimalarial drug. Gilmore and co-workers have presented a scheme where artemisinic acid from *A. annua* and genetically modified yeast can be converted into β-artemether, β-artemotil and artesunate (Figure 4) [69]. This whole process is comprised of different modules or reaction steps including photooxidation/cyclization, reduction, and derivatization and continuous purification [69]. The side products formed during the production of artemisinin should also be converted into useful derivatives that have antimalarial properties. Through such methods, the production of active ingredients from the *Artemisia annua* can be increased.

Beside the tobacco plant, mosses could also be used for the production of artemisinin. In case of mosses, *Physcomitrella patens* are used as hosts for the production of artemisinin and other valuable natural products [70,71]. The advantages of *P. patens* are that, the rate of homologous recombination is high; its genome is fully sequences and several in vivo studies for DNA fragments assembly have already been performed [70,71]. It has been reported that *P. patens* produces three commercially important sesquiterpenoids that include patchoulol, β-santalene, and sclareol in milligrams per gram of their dry weight [72,73]. Recently, Ikram et al. introduced all the five important artemisinin pathway genes into *P. patens* through multiple DNA fragment-based methodology [70,71]. This new transgenic *P. patens* make approximately 0.21 mg/g of it dry weight of artemisinin [70,71]. The authors reported that the intermediate competing pathways of other natural products are also absent in *P. patens* [70,71].

The genetically engineered cyanobacteria and algae are also green factories for the production of different biofuels, antibodies, nutraceuticals and other beneficial products [74,75,76,77,78,79]. The advantage of growing cyanobacteria and algae is that it can be harvested in five to six days, they grow phototrophically in water [74,75,76]. The cyanobacteria and algae cells also contain a large chloroplast like a cell from the leaf of tobacco plant. Thus, growing them in large ponds for the production of artemisinin is a better choice. Recently, *Synechococcus elongatus* PCC 7942 was bioengineered for the production amorpha-4,11-diene by modulating the metabolic pathway of amorphadiene synthase and methylerythritol phosphate pathway enzymes [80]. This bioengineered cyanobacteria produced 23-times higher (19.8 mg/L) content of amorpha-4,11-diene as compared to the wild type [80]. The amorpha-4,11-diene is a precursor of artemisinin. Thus, through bioengineering, we can modify cyanobacteria for the production of low-cost and high-content artemisinin. The use of cyanobacteria and algae farms for the production of artemisinin will definitely lower the cost of the drug and the rate of production will also be high. However, the problem is that of contamination by wild cyanobacteria and algae. The risks of failure are always there, but with the increase in algae farming and its growth on commercial basis by companies like algenol, Sapphire Energy and dozens of others, it is important that they should also try the generation of artemisinin from algae, in the same way they are producing nutraceuticals like astaxanthin, etc.

## 3. Advancements in Understanding the Mode of Action of Artemisinin and Related Drugs

The mode of action of artemisinin and its derivatives is debatable and a number of studies have been performed to elucidate the actual mechanism of action [81,82]. Both oxidative stress and alkylation of heme along with plasmodial proteins are considered in the mode of action of artemisinin and its related compounds [83]. One study suggested that the heme from the parasite heme synthesis pathway is alkylated along with other important parasite proteins and is hence an inhibitor of plasmodium growth [84]. A proteomic technique has provided first-hand information about the targeted proteins of asexual erythrocyte stage of plasmodium that are alkylated by the 1,2,4-trixolane group of antimalarial compounds [85]. These alkylated proteins are part of glycolysis, host hemoglobin splitting, antioxidant defense, protein synthesis and stress pathways that are extremely important for the parasite persistence [85]. The same approach is used for the action of artemisinin on various target proteins [86]. The in situ analyses showed that the alkylated proteins are involved in parasite glycolysis, catabolism of hemoglobin, redox function and biosynthesis of other proteins [86]. These biochemical approaches are helpful in determining the different modes of action of antimalarial drugs. Furthermore, these results provided clues about the fate of the alkylated proteins of the protozoan. In another experimental setup, the free radicals generated due to the reaction of artemisinin and its derivatives were monitored through electron spin resonance (ESR), a combination of high performance liquid chromatography–electron spin resonance (HPLC-ESR) and a combination of high performance liquid chromatography–electron spin resonance-mass spectrometry (HPLC-ESR-MS) [87]. The free radicals were monitored chemically with the help of Fe^2+^ while α-(4-Pyridyl-1-oxide)-*N*-*tert*-butylnitrone (4-POBN) was used as a spin trap during the reactions [87]. During the measurements, a number of radicals were detected; thus, these radicals exert their action on the parasite and destroy it [87]. Artmisinin also acts as an allelochemical that inhibit the growth of different plants seedling [88]. When the plants seeds are treated with artemisinin they died due to oxidative stress through the generation of reactive oxygen species (ROS) [88]. These ROS performed lipid peroxidation, inhibition of mitosis and ultimately caused cell death [88]. It has also been suggested that the ROS damage the parasite Ca^2+^-ATPase enzyme that is responsible for calcium ion transport [89]. In the artemisinin-resistant plasmodium species, a PfATP6 L263 mutation is also normally observed [89].

## 4. Marine Sponges as a Source of Endoperoxides

**Figure 5:**
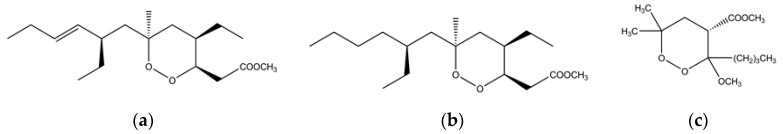
Chemical structure of (**a**) plaktorin; (**b**) dihydroplaktorin and (**c**) 3-methoxy-1,2-dioxane synthetic analogue.

Marine sponges, especially Plakortis, are a rich sources of natural secondary metabolites and several effective antiplasmodial agents have been isolated from them in the past few years [90,91]. For example, a thiazine-containing alkaloid known as thiaplakortone A is present in *Plakortis lita*, a native of Australia [90,91]. The thiaplakortone A is equally efficacious against chloroquine-resistant and chloroquine-sensitive *P. falciparum* [91,92]. The search of antimalarial compounds in the marine world led to the identification of endoperoxides (1,2-dioxanes)-based polyketides in a Caribbean sponge named *Plakortis simplex* [93]. These 1,2-dioxane-containing polyketides are plakortin and dihydroplakortin (Figure 5) and are potent antimalarial agents in nanomolar concentrations in vitro against chloroquine-resistant *P. falciparum,* and in vivo against *P. berghei* in murine models without any observed toxicity [93]. Inspired by the plakortin, a synthetic compound has also been prepared and it works in a similar fashion like the natural metabolites [93]. These 1,2-dioxanes-based natural and synthetic polyketides produce carbon-based free radicals when they interact with the heme Fe(II) [93]. Like in the case of artemisinin, the ferrous catalyzed reductive cleavage of the endoperoxide of plakortin and its derivatives produce radicals that are toxic to the parasite [93,94]. In a number of other studies, it was observed that different species of plaktoris sponge are a rich source of cyclic and linear peroxides that have antimalarial, antifungal and anticancer properties [95,96,97,98,99,100,101,102,103].

## 5. Artemisinin Inspired Novel Antimalarial Compounds

**Figure 6:**
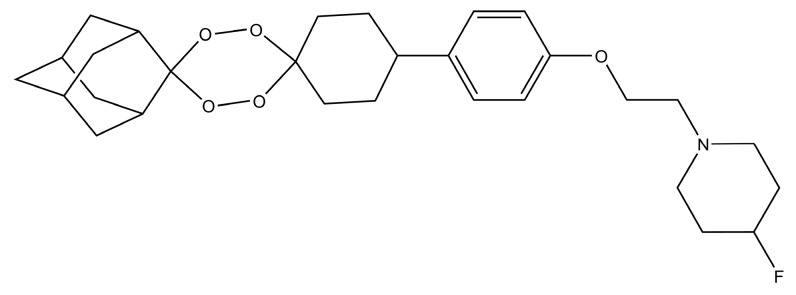
Chemical Structure of E209 compound.

The K13 gene mutations which confer resistance against artemisinin to *P. falciparum* are the main threat to artemisinin-based therapy for malaria treatment [104]. Thus, most of the antimalarial drug efforts are to design vaccines and compounds which can target such resistant plasmodium species [104]. Furthermore, isolation from *A. annua* is the only practical source of artemisinin, whose annual growth affects the drug supply and cost [105]. Another shortcoming of artemisinin and its derivatives is their short in vivo half-lives, therefore, there is a need of novel synthetic antimalarial drugs [105]. Recently, due to the efforts of a multinational antimalarial efforts, a tetraoxane-based molecule named E209 has been synthesized (Figure 6) [104]. The E209 has a potency in nanomolar concentrations against different strains of *P. falciparum* and *P. vivax* both in vitro and in vivo [104]. E209 is equally efficient as dihydroartemisinin against malarial parasites, has similar pharmaceutical properties and is capable of a single-dose cure [104]. Thus, in the future, endoperoxide-based compounds like E209 will be used for artemisinin resistance Plasmodium. Similarly, other natural product compounds are also being investigated by different authors for their antimalarial properties [106].

**Figure 7:**
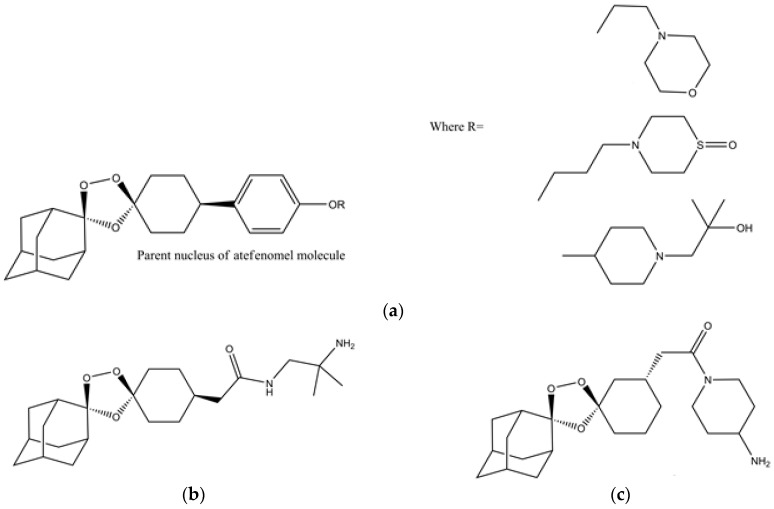
(**a**) Chemical structure of artefenomel and its optimized tertiary amine analogues [105]; (**b**) arterolane; (**c**) arterolane analogue that has higher antiplasmodial activity than arterolane [115].

Artefenomel (OZ439) (Figure 7a) is a synthetic trioxolane that possesses the artemisinin pharmacophore and has enhanced pharmacokinetic properties [107,108]. Artefenomel is quite efficacious and has prolonged blood concentrations due to its stabilizing power to protect the unstable peroxide bond, thus, it has proper retention time in plasma and also produces the required ferrous reactivity to terminate the plasmodium [109]. Currently, it is synthesized on industrial scales in india and it is also an approved antimalarial drug in seven malaria endemic African countries [108]. Its level of tolerance is around 1600 mg in different volunteers [107]. Clinical trials in volunteers from Thailand showed that it has the capacity to control uncomplicated malaria caused by *P. falciparum* and *P. vivax* [107]. Furthermore, there were no sides effects observed during these clinical trials [107]. The authors suggested its use in combination with other drugs like ferroquine, piperaquine and DSM265 [107]. The arterolane and artefenomel are more effective against K13 mutant of *P. falciparum* as compared to dihydroartemisinin [110]. However in another observation it was noted that the dihydroartemisinin is more potent than the ozonides like OZ439 and OZ277 when they are used for the treatment of K13 mutants in asexual erytrocytic stage protozoans [111]. These results showed that the K13 mutant strains may be a graveyard for antimalarial drugs in the future, if they are not used with caution. In recent times, the industrial scale production of artefenomel is an alternative to artemisinin in ACTs. Thus, in the future, the demand for these synthetic endoperoxide drugs will increase due to the spread of K13 resistant strains of plasmodium in different Asian countries.

The structure–activity relationship studies of ozonide artefenomel show that the primary and secondary amino ozonides have increased metabolic stability due to their high pKa and lower Log D7.4 values [112]. Attaching a polar group to the primary and tertiary amino ozonides resulted into a decrease in in vivo antimalarial activity [112]. On the other hand, adding a cycloalkyl and heterocyclic groups to the primary and tertiary amino ozonides resulted in higher antimalarial potency than adding an acyclic group to them [112]. This increase in potency is due to an increase in plasma retention time [112]. Thus, antimalarial drugs with longer plasma retention times are suitable for the control of erytrocytic stage plasmodium.

It has been proposed that, like artemisinin, the antimalarial synthetic peroxides also undergo reductive cleavage by the ferrous heme of the catabolized hemoglobin [113]. This peroxide reduction resulted in active carbon-centered radicals that alkylate heme and the parasite proteins [113]. In order to understand the proposed mechanism of action of famous Ozonides OZ277 (arterolane) (Figure 7b) and OZ439 (artefenomel), monoclonal antibodies are used to probe the alkylation of heme and plasmodial proteins [113]. From the Immunofluorescence analysis of ozonide-treated parasitic proteins and heme, it was observed that only *P. falciparum* proteins are alkylated while there is no action on the host heme proteins [113]. These experiments proved that there is no cross reactivity between the endoperoxide antimalarial drugs and host proteins. Thus, the endoperoxides drugs have lower toxicity and have high selectivity and specificity.

In a related SAR investigation, it was observed that tertiary amine-containing ozonides are less toxic as compared to the primary and secondary amine-containing ozonides (Figure 7a) [105]. They have suitable pharmacological profiles and are potent against *P. berghei* in murine models [105]. They can be easily prepared through the Griesbaum co-ozonolysis scheme with low cost [105]. It has been observed that making modifications in bond rigidity, adding different groups like amines, etc., can increase the potency against the resistant species [114]. It was also noted that changing the stereochemistry and position of different groups attached to the nucleus of drugs can enhance the antiplasmodial efficacy [115]. For example a stereoisomeric analogue (Figure 7c) of arterolane is more potent than the arterolane itself [115].

## 6. Conclusions and Future Perspectives

The eradication of malaria will eventually need an integrated strategy that includes the combination of new and old drugs, vector control, and use of vaccines and to take steps in public health. Until now the elimination task of the malaria seems discouraging because the old strategies like spraying indoor and use of nets are not sufficient, especially in the endemic regions, therefore, only innovative scientific discoveries can change the situation. Expression of the whole biosynthetic pathway of artemisinin enzymes in tobacco plants will lead to a high production of artemisinin at low cost. This is an exemplary work which will control malaria in third world countries as now patients will be able to afford the drug, but for such a dream to come true, it is the responsibility of government agencies, farmers and pharama companies to collaborate with one another and make this goal of a malaria-free world achievable. This method of expression of the whole biosynthetic pathway of foreign enzymes in tobacco can be exploited to express other medicinally important alkaloids, and thus, the cost of various drugs can be lower down. Similarly work on synthetic endoperoxides should also be expedited, as they provide an alternative way to lower the cost of the antimalarial drugs and for the control of artemisinin resistant plasmodium species.

## Abbreviations

ACTs	Artemisinin-based combination therapies
COSTREL	combinatorial super transformation of transplastomic recipient lines
ALDH1	aldehyde dehydrogenase
DXR	1-deoxy-d-xylulose-5-phosphate reductoisomerase
ELQ	Endochin-like quinolones
